# Geriatric Nutritional Risk Index Predicts High Activities of Daily Living at Discharge in Older Patients with Heart Failure after Cardiac Rehabilitation

**DOI:** 10.3390/jcm12247662

**Published:** 2023-12-13

**Authors:** Yuta Muraoka, Takahiro Miura, Midori Miyagi, Tatsuma Okazaki, Taiki Katsumata, Keisuke Obata, Satoru Ebihara

**Affiliations:** Department of Internal Medicine and Rehabilitation Science, Tohoku University Graduate School of Medicine, Sendai 980-8574, Japan; cloverbox920@yahoo.co.jp (Y.M.); takahiro.miura.d1@tohoku.ac.jp (T.M.); midori.miyagi.b5@tohoku.ac.jp (M.M.); tatsuma.okazaki.c5@tohoku.ac.jp (T.O.); katsumata.taiki.q4@dc.tohoku.ac.jp (T.K.); keisuke.obata1110@gmail.com (K.O.)

**Keywords:** high ADL at discharge, geriatric nutritional risk index, heart failure, cardiac rehabilitation

## Abstract

Background: Hospitalization often leads to a decline in activities of daily living (ADL) in older patients with heart failure. Although cardiac rehabilitation (CR) improves ADL, it can be difficult to perform CR due to the deconditioning of these patients. This study aimed to examine the factors associated with ADL at discharge in older patients with heart failure who underwent CR. Methods: A total of 86 of 110 older heart failure patients aged ≥ 75 years (average age, 86.9 ± 5.7 years) transferred to our institution for CR were enrolled and classified into high ADL at discharge (*n* = 54) and low ADL at discharge (*n* = 32) groups. Physical characteristics, comorbidities, medications, blood test data, echocardiographic data, and nutritional status (Geriatric Nutritional Risk Index [GNRI]) were retrospectively examined from medical records. ADL were assessed using the Barthel Index (BI) at admission and discharge. Considering multicollinearity, the relationship between high ADL (BI ≥ 60) at discharge and these assessments at admission was analyzed using multiple logistic regression analysis. The receiver operating characteristic curve was analyzed to calculate the cutoff values for the parameters identified by the multiple logistic regression analysis. Results: The GNRI was the only independent factor predicting high ADL at discharge (*p* = 0.041; odds ratio [OR], 1.125; 95% confidence interval [CI], 1.005–1.260). The area under the receiver operating characteristic curve for the GNRI was 0.770 (95% CI, 0.664–0.876). The cutoff value for the GNRI was 83.4 (sensitivity, 85.2%; specificity, 62.5%). Conclusion: These findings suggest that the GNRI score at admission predicts high ADL at discharge in older patients with heart failure who underwent CR.

## 1. Introduction

The increase in the incidence of heart failure in the older population is a global problem [1], which is accompanied by an increased prevalence of multimorbidity [2,3]. Many older patients with heart failure require physical and social care in addition to disease management [2,3]. Therefore, activities of daily living (ADL) tend to decline before hospitalization, and proceed to decline after hospitalization due to bed rest [4]. Low ADL, particularly an impaired Barthel Index (BI < 60), is closely associated with a poor prognosis [3,4,5], but inpatient cardiac rehabilitation (CR) improves ADL in older patients with heart failure [3,6]. However, it can be difficult to perform ordinary inpatient CR due to the deconditioning of these patients; therefore, their exercise prescriptions involve very low-intensity and intermittent training [7]. As a result, it can be difficult to achieve high ADL scores at discharge in older patients with heart failure.

Local edema of the intestinal wall in patients with heart failure may contribute to malabsorption [8]. Additionally, sympathetic nerve activity and appetite loss in heart failure patients increase in proportion to the severity of heart failure [9,10]. Therefore, heart failure patients are prone to malnutrition. Recently, many older patients with heart failure have had a high risk of malnutrition. A recent study found that 46.5% of hospitalized older patients with heart failure exhibited signs of malnutrition [11]. Additionally, poor nutritional status is an independent poor prognostic factor. Low body weight due to malnutrition was associated with increased mortality in older patients with heart failure [12,13]. Therefore, the clinical importance of nutritional management in these patients has increased. Kinugasa et al. suggested that nutritional status is associated with ADL [14]. Studies have addressed the relationship between nutritional status and ADL in older patients with heart failure who underwent CR [15,16]; however, the association between nutritional status and a high ADL (BI ≥ 60) remains unreported.

This study aimed to examine the factors associated with high ADL scores at discharge in older patients with heart failure.

## 2. Materials and Methods

### 2.1. Participants

This retrospective cohort study recruited 110 older patients with heart failure aged ≥ 75 years who transferred to our institution for CR between 1 April 2015 and 31 July 2022. Our institution accepts patients with stable conditions who have been treated at acute care hospitals. Inclusion criteria were as follows: (1) patients with heart failure who transferred to our institution for CR, and (2) patients aged ≥ 75 years. Exclusion criteria were as follows: (1) in-hospital mortality, (2) missing data, (3) undergoing surgical treatment, and (4) difficulty consenting to participate. Among the 110 recruited patients, 24 were excluded owing to in-hospital mortality (*n* = 16) and missing data (*n* = 8), and 86 patients were eventually included in the study. Heart failure was diagnosed by a cardiologist based on the criteria of the Japanese Circulation Society guidelines (JCS 2017/JHFS 2017) for the Guideline on Diagnosis and Treatment of Acute and Chronic Heart Failure [17].

**Figure 1:**
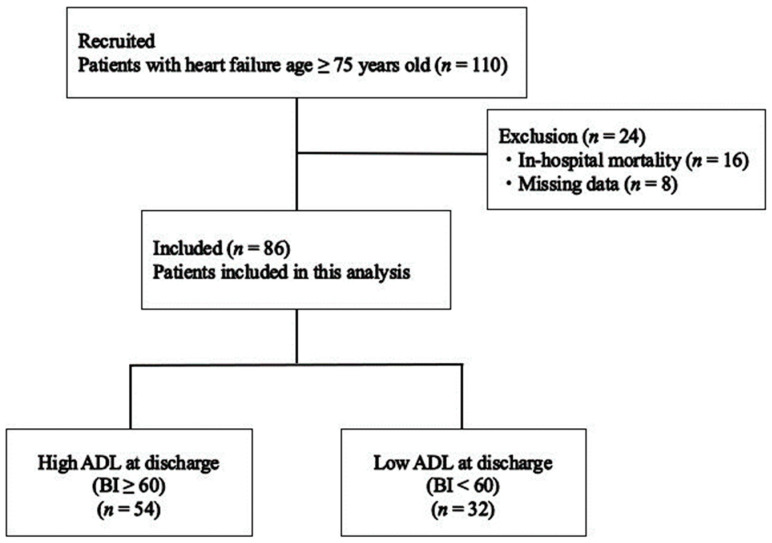
Flowchart of study participants. BI—Barthel Index.

These patients were referred for inpatient late phase II CR by a cardiologist after examination at admission and contraindications to exercise therapy were confirmed. The primary physician determined the prescription and timing of CR. CR at our institution is recommended for all older patients with heart failure. CR was performed according to the guidelines of the Japanese Circulation Society for the rehabilitation of inpatients with cardiovascular disease (JCS 2012, JCS 2021) [7,18]. The participants were divided into two groups based on their ADL scores at discharge using the BI. Patients who had high ADL (BI ≥ 60) and low ADL (BI < 60) scores at discharge were classified into the high ADL (*n* = 54) and low ADL groups (*n* = 32). The evaluation parameters were retrospectively examined from medical records. A flowchart of the inclusion of participants is shown in Figure 1.

### 2.2. Evaluation Parameters

Physical characteristics including age, sex, prior heart failure hospitalization, body mass index (BMI), length of hospital stays, nursing-care insurance, New York Heart Association (NYHA) functional class, comorbidities, medication data, blood test data, and echocardiographic data at admission were retrospectively examined from medical records. Comorbidities were examined for coronary artery disease, hypertension, atrial fibrillation, diabetes, valvular disease, cardiomyopathy, respiratory disease, cerebrovascular disease, dyslipidemia, chronic kidney disease (CKD), and dementia. Medication data were examined for angiotensin-converting enzyme inhibitor (ACEI), angiotensin II receptor blocker (ARB), diuretics, β-blockers, and angiotensin receptor neprilysin inhibitor (ARNI). Blood test data were examined for brain natriuretic peptide (BNP), hemoglobin (Hb), blood urea nitrogen (BUN), creatinine (Cre), estimated glomerular filtration rate (eGFR), albumin (Alb), total protein (TP), total cholesterol (TC), triglyceride (TG), high-density lipoprotein (HDL-C), low-density lipoprotein (LDL-C), and C-reactive protein (CRP). Echocardiographic data were examined for left atrial dimension (LAD) and left ventricular ejection fraction (LVEF). ADL scores at admission and discharge were retrospectively assessed from medical records using the BI.

In this study, BNP was measured as N-terminal proBNP (NT-proBNP) is more affected by decline in renal function than BNP [19]. Additionally, the medical costs of NTproBNP are higher than those of BNP.

The BI is a 10-item scoring assessment scale used to measure a patient’s functional ability to perform basic activities of daily living [20]. The BI includes self-care independence (feeding, grooming, bathing, dressing, bowel and bladder care, and using the toilet) and mobility independence (ambulation, transferring, and going up and down stairs), with a score of 0 (completely dependent) to 100 points (completely independent). In this study, BI scores were assessed by a physical therapist.

The cutoff value of the BI used to divide the patients into groups was 60, based on a previous study demonstrating poor prognosis in older patients with heart failure who underwent CR [5].

Nutritional status was also retrospectively assessed based on medical records at admission using the Geriatric Nutritional Risk Index (GNRI). This nutritional scale precisely predicts mortality rates in older people [21]. The GNRI score was calculated using serum albumin and BMI at admission and assessed using the following formula:

GNRI = [14.89 × serum Alb value (g/L)] + [41.7 × (actual weight/reference weight)]

Reference weight (kg) = (height)^2^(m^2^) × 22

The reference (ideal) weight was set as the weight resulting in a BMI of 22 kg/m^2^. When the actual weight exceeded the reference weight, actual weight/reference weight was calculated as 1. The correlation coefficient between the GNRI calculated by the ideal BMI of 22, and that calculated by the Lorentz formula, is high [22,23]. The GNRI scores were reported as 4 grades of nutrition-related risk: major risk (GNRI < 82), moderate risk (GNRI: 82 to <92), low risk (GNRI: 92 to ≤98), and no risk (98 < GNRI) [21]. In this study, the score of GNRI < 92 was defined as low GNRI based on a previous study [21].

### 2.3. Statistical Analysis

To identify factors associated with high ADL (BI > 60) at discharge, continuous variables, including physical characteristics, blood test data, echocardiographic data, and GNRI at admission, were compared using an unpaired t-test for normally distributed variables and the Mann–Whitney U-test for non-normally distributed variables. Categorical variables were compared using chi-square and Fisher’s exact tests, as required. Multiple logistic regression analysis using the forced-entry method was used to assess the effect of factors associated with high ADL at discharge. A high ADL score at discharge was used as the dependent variable. Parameters with statistically significant differences between the two groups were set as explanatory variables.

A receiver operating characteristic (ROC) curve analysis was performed on the parameters identified via multiple logistic regression to calculate cutoff values, sensitivity, and specificity using the Youden index. Statistical analysis was performed using SPSS (IBM Corp. Released 2010. IBM SPSS Statistics for Windows, Version 19.0. Armonk, NY, USA: IBM Corp.) and JMP (JMP^®^, Version 16. SAS Institute Inc., Cary, NC, USA, 1989–2023.), with significance set at *p* < 0.05.

## 3. Results

The baseline characteristics of the participants at admission are shown in Table 1. The cohort had a mean age of 86.9 ± 5.7 years, with 34.9% being men, 32.6% having prior heart failure hospitalization, a mean BMI of 20.3 (18.7, 23.1), 61.6% having NYHA functional class III/IV, and an admission BI score of 43.2 ± 25.8. Overall, 46.5% of the participants had coronary artery disease, and the most common comorbidity was dyslipidemia (72.5%), followed by hypertension (69.8%) and atrial fibrillation (51.2%). A total of 7% of the participants had cardiomyopathy. The cohort included hypertrophic cardiomyopathy (*n* = 2), ischemic cardiomyopathy (*n* = 2), and drug-induced cardiomyopathy (*n* = 2). ACEI/ARB, diuretics, and β-blockers and ARNI were prescribed to 59.3%, 79.1%, 68.6%, and 5.8% of the patients, respectively. The median LVEF was 60.8 (47.2, 69.2). The cohort comprised 12 patients with heart failure with reduced ejection fraction (HFrEF), 12 patients with heart failure with mid-range ejection fraction (HFmrEF), and 62 patients with heart failure with preserved ejection fraction (HFpEF) based on the guidelines, respectively [17]. 

Significant differences were observed between the high and low ADL groups in terms of age, sex, BMI, NYHA functional class III/IV, admission BI scores, dyslipidemia, Cre levels, eGFR levels, Alb levels, TP levels, CRP levels, and admission GNRI scores (Table 1).

Pearson’s product–moment correlation coefficient was used to account for multicollinearity. The correlation coefficient for the GNRI score and Alb levels was *r* = 0.913 (*p* < 0.0001). The correlation coefficient for eGFR and Cre levels was *r* = −0.811 (*p* < 0.0001). The correlation ratios between high ADL at discharge and factors showing multicollinearity were *η* = 0.479 for the GNRI, *η* = 0.395 for Alb levels, *η* = 0.270 for eGFR, and *η* = 0.250 for Cre levels. Therefore, the GNRI and eGFR were selected as explanatory variables for the multiple logistic regression analysis.

Table 2 shows the results of the multiple logistic regression analysis used to examine the association between high ADL at discharge and the evaluation parameters associated with it. The multiple logistic regression analysis was performed using the GNRI, BI, age, sex, BMI, NYHA functional class III/IV, dyslipidemia, eGFR, CRP, and TP levels as explanatory variables. GNRI was identified as the only independent factor predicting high ADL at discharge (*p* = 0.041; odds ratio [OR], 1.125; 95% confidence interval [CI], 1.005–1.260). The model χ^2^ test result was significant at *p* < 0.01.

**Figure 2:**
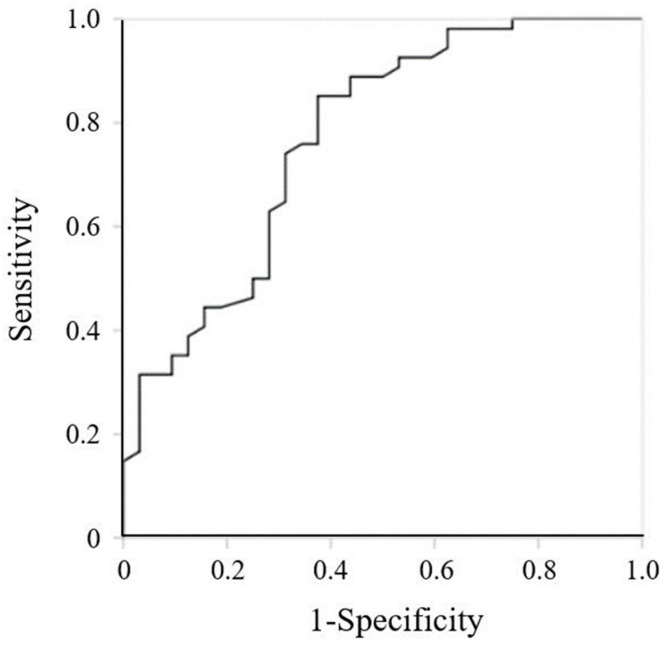
Receiver operating characteristic curve for Geriatric Nutritional Risk Index at admission.

Figure 2 shows the ROC curve for the GNRI. The area under the curve was 0.770 (95% CI, 0.664–0.876, *p* < 0.001). The cutoff value for the GNRI was 83.4 (sensitivity, 85.2%; specificity, 62.5%).

Table 3 shows the results of the main parameters according to LVEF classification. No differences were observed between groups.

## 4. Discussion

This is the first study to demonstrate the association between nutritional status and ADL at discharge in older patients with heart failure aged ≥ 75 years who underwent CR. The GNRI specifically was identified as a factor associated with high ADL at discharge; thus, malnutrition at admission is an independent risk factor for low ADL at discharge. These findings provide valuable insights for achieving high ADL scores at discharge and promoting CR in older patients with heart failure.

Factors associated with the pathophysiology and treatment of heart failure, such as cardiopulmonary failure, bed rest, myopathy, and malnutrition, may cause and exacerbate muscle weakness, leading to decreased ADL [14,24]. The quadriceps muscle mass (the major muscle of knee extension) decreased by approximately 12.5% after only 7 days of bed rest [25]. In addition, knee extension strength, which is essential for independent ambulation and walking, positively correlates with ADL [26]. In the present study, a low GNRI score at admission was identified as a factor associated with low ADL (BI < 60) at discharge in older patients with heart failure who underwent cardiac rehabilitation. Thus, muscle weakness resulting from the pathophysiology, treatment background, and malnutrition may have led to a decline in the ADL of the patients.

Malnutrition in patients with heart failure tends to result from gut malabsorption, appetite loss, catabolic and anabolic imbalance, and physiological changes due to aging [7,27]. A study proposed that increased intestinal wall edema translocates bacterial endotoxins from the intestine, ultimately leading to the production of proinflammatory cytokines from monocytes in the bloodstream [28]. Consequently, catabolism is exacerbated by increased levels of these proinflammatory mediators. Reduced intestinal circulation may contribute to local edema and malabsorption in the intestinal wall [8]. The causes of appetite loss include proinflammatory cytokines, gastrointestinal congestion, and gastrointestinal dysfunction [27,29]. Thus, older patients with heart failure may develop malnutrition based on this pathophysiology.

Early initiation of feeding may maintain high ADL in older patients with heart failure [30]. However, the mean age of patients in our study was higher than that in the previously mentioned study. Exercise training and nutritional intervention improve muscle strength and energy intake in older people with various comorbidities (mean age 87 years) [31]. In our study, GNRI at admission was identified as a factor associated with high ADL (BI ≥ 60) at discharge in older patients with heart failure who underwent cardiac rehabilitation; thus, in addition to cardiac rehabilitation, nutritional intervention from the time of admission may be important to achieve high ADL at discharge.

Recently, the prevalence of HFpEF in older patients has gradually increased with age and is higher in women than in men of all ages [32]. The severity of HFpEF increases more rapidly with age than that of HFrEF [33]. Additionally, non-cardiac comorbidities are highly prevalent in patients with HFpEF and can induce a systemic inflammatory state [34,35]. Therefore, the elevated levels of circulating inflammatory biomarkers were more pronounced in patients with HFpEF than in those with HFrEF. The control of proinflammatory pathways has been associated with reduced severity and improved outcomes in patients with HFpEF [33]. Studies suggest that exercise training and nutritional support improve the levels of inflammatory markers in patients with heart failure [36,37]; therefore, they are necessary interventions.

The cutoff value of GNRI < 92 is commonly used to assess the risk of morbidity and mortality in hospitalized older patients [21]. In this study, the cutoff value was 83.4 for high ADL (BI ≥ 60) at discharge, giving a sensitivity of 85.2% and specificity of 62.5%. A relationship between nutritional status and continuous walking distance has been reported [38]. While heart failure patients with a mean GNRI ≥ 92 could walk at least 50 m, those who have a mean GNRI of 87 ± 10 could only walk within 50 m. Ambulatory independence is defined as being able to walk on level ground for more than 45 m [20]. Additionally, Shah et al. reported that a BI < 60 indicates a severe decline in function [39]. Thus, most patients in the present study may not have been capable of walking independently, considering the relationship between the low GNRI score and decline in ADL. 

Previous studies reported that the mean age of hospitalized older patients with heart failure was 78.0 ± 12.5 years, and most older patients with heart failure in Japan were older than 75 [40]. The distribution of older patients hospitalized with heart failure peaked in the 80–89-year-old age group for both men and women [3]. Additionally, the median ADL score based on the BI at admission was as low as 25 (0–80), and the proportion of patients capable of walking and using the toilet independently was 37.6% and 29.3%, respectively [3]. In this study, the mean age and mean ADL at admission of the overall cohort were 86.9 ± 5.7 years and 43.2 ± 25.8, respectively, and there were more women (65.1%) than men. Thus, this study reflects the real-world clinical data of older patients with heart failure.

This study has several limitations. First, this was a single-center retrospective observational study with a small sample size. Second, the leg muscle strength and frailty were not assessed. Third, this study was insufficient for assessment at discharge and did not assess changes in GNRI scores. Therefore, further multicenter and prospective studies with a large sample size are required.

In conclusion, the present study showed that malnutrition at admission reduced ADL at discharge (BI < 60) in older patients with heart failure who underwent cardiac rehabilitation. Appropriate nutritional intervention is important for high ADL at discharge in these patients and may lead to an improved prognosis.

## Figures and Tables

**Table 1:** Baseline characteristics of the high ADL and low ADL groups.

	Overall (*n* = 86)	High ADL (*n* = 54)	Low ADL (*n* = 32)	*p*-Value
Age (years)	86.9 ± 5.7	85.8 ± 5.6	88.8 ± 5.6	0.0170
Male, *n* (%)	30 (34.9)	14 (25.9)	16 (50.0)	0.0236
Prior HF hospitalization, *n* (%)	28 (32.6)	17 (31.5)	11 (34.4)	0.7819
Nursing-care insurance, *n* (%)	55 (64.0)	34 (63.0)	21 (65.6)	0.8037
BMI (kg/m^2^)	20.3 (18.7, 23.1)	21.4 (19.1, 24.1)	19.1 (17.3, 21.5)	0.0044
Length of hospital stays (days)	53.0 (32.0, 81.5)	44.0 (28.0, 70.5)	62.0 (37.5, 96.0)	0.0553
NYHA class III/IV, *n* (%)	53 (61.6)	25 (46.3)	28 (87.5)	0.0001
Exacerbation of HF, *n* (%)	28 (32.6)	14 (25.9)	14 (43.8)	0.0880
Intravenous diuretics, *n* (%)	10 (11.6)	5 (9.3)	5 (15.6)	0.2890
Admission BI (score)	43.2 ± 25.8	53.1 ± 23.7	26.4 ± 20.1	*p* < 0.0001
Comorbidity				
Coronary artery disease, *n* (%)	40 (46.5)	27 (50.0)	13 (40.6)	0.3995
Hypertension, *n* (%)	60 (69.8)	37 (68.5)	23 (71.9)	0.7432
Atrial fibrillation, *n* (%)	44 (51.2)	26 (48.1)	18 (56.3)	0.4675
Diabetes, *n* (%)	27 (31.4)	19 (35.2)	8 (25.0)	0.3252
Valvular disease, *n* (%)	40 (46.5)	22 (40.7)	18 (56.3)	0.1634
Cardiomyopathy, *n* (%)	6 (7.0)	6 (11.1)	0 (0.0)	0.0506
Respiratory disease, *n* (%)	21 (24.4)	12 (22.2)	9 (28.1)	0.5380
Cerebrovascular disease, *n* (%)	19 (22.1)	11 (20.4)	8 (25.0)	0.6169
Dyslipidemia, *n* (%)	58 (72.5)	46 (82.1)	12 (50.0)	0.0032
CKD, *n* (%)	10 (11.6)	8 (14.8)	2 (6.3)	0.2311
Dementia, *n* (%)	20 (23.3)	11 (20.4)	9 (28.1)	0.4106
Medication				
ACEI/ARB, *n* (%)	51 (59.3)	34 (63.0)	17 (53.1)	0.3690
Diuretics, *n* (%)	68 (79.1)	44 (81.5)	24 (75.0)	0.4751
β-blockers, *n* (%)	59 (68.6)	37 (68.5)	22 (68.8)	0.9822
ARNI, *n* (%)	5 (5.8)	4 (7.4)	1 (3.1)	0.3810
Blood test data				
BNP (pg/mL)	356.6 (182.7, 771.3)	414.6 (213.2, 819.1)	329.0 (158.5, 730.6)	0.4396
Hb (g/dL)	10.7 ± 1.6	10.7 ± 1.7	10.7 ± 1.5	0.8465
BUN (mg/dL)	23.8 (18.2, 30.2)	23.1 (16.9, 30.7)	23.8 (18.9, 29.5)	0.8899
Cre (mg/dL)	0.98 (0.75, 1.31)	1.08 (0.79, 1.46)	0.89 (0.72, 1.10)	0.0342
eGFR (mL/min/1.73 m^2^)	45.6 (32.4, 59.9)	39.15 (30.2, 55.5)	54.6 (39.8, 73.1)	0.0084
Alb (g/dL)	3.3 ± 0.5	3.5 ± 0.5	3.1 ± 0.5	0.0002
TP (g/dL)	6.7 ± 0.7	6.8 ± 0.6	6.5 ± 0.8	0.0343
TC (mg/dL)	161.4 ± 35.4	161.4 ± 37.3	161.4 ± 32.7	0.9931
TG (mg/dL)	87.5 (73.5, 132.0)	87.0 (71.0, 137.3)	88.5 (78.5, 120.5)	0.7308
HDL-C (mg/dL)	46.0 (40.0, 56.0)	48.5 (41.0, 54.5)	44.0 (37.0, 57.5)	0.2714
LDL-C (mg/dL)	93.5 (71.3, 116.0)	92.5 (71.3, 108.5)	98.0 (70.3, 120.5)	0.3812
CRP (mg/dL)	0.56 (0.12, 1.81)	0.29 (0.08, 1.40)	0.78 (0.29, 2.85)	0.0429
Echocardiographic				
LAD (mm)	40.8 ± 9.0	41.9 ± 8.5	39.1 ± 9.6	0.1562
LVEF (%)	60.8 (47.2, 69.2)	60.3 (47.5, 70.1)	61.6 (46.1, 68.9)	0.9537
Nutrition				
GNRI	87.6 ± 9.2	91.0 ± 7.6	82.0 ± 8.9	*p* < 0.0001

ADL—activities of daily living; BI—Barthel Index; BMI—body mass index; HF—heart failure; NYHA—New York Heart Association; ACEI—angiotensin-converting enzyme inhibitor; ARB—angiotensin II receptor blocker; ARNI—angiotensin receptor neprilysin inhibitor; LAD—left atrial dimension; LVEF—left ventricular ejection fraction; BNP—brain natriuretic peptide; Hb—Hemoglobin; BUN—blood urea nitrogen; Cre—creatinine; eGFR—estimated glomerular filtration rate; Alb—albumin; TP—total protein; TC—total cholesterol; TG—triglyceride; HDL-C—high-density lipoprotein; LDL-C—low-density lipoprotein; CRP—C-reactive protein; GNRI—Geriatric Nutritional Risk Index; CKD—chronic kidney disease.

**Table 2:** Multiple logistic regression analysis regarding factors associated with high ADL at discharge.

Variable			95% CI	
	*p*-Value	Odds Ratio	Lower Limit	Upper Limit
Admission BI (score)	0.299	1.020	0.983	1.058
Age (years)	0.088	0.890	0.779	1.017
Male, *n* (%)	0.177	2.491	0.661	9.385
BMI (kg/m^2^)	0.708	1.038	0.855	1.261
GNRI	0.041 *	1.125	1.005	1.260
NYHA class III/IV, *n* (%)	0.063	5.729	0.909	36.121
Dyslipidemia, *n* (%)	0.261	0.469	0.126	1.754
eGFR (mL/min/1.73 m^2^)	0.140	0.974	0.941	1.009
CRP (mg/dL)	0.187	1.295	0.894	1.772
TP (g/dL)	0.574	0.730	0.243	2.189

Model χ^2^-test: *p* < 0.01; Hosmer–Lemeshow test: *p* = 0.380; discriminative predictive value: 83.7%. CI, confidence interval. * *p* < 0.05. BI—Barthel Index; BMI—body mass index; GNRI—Geriatric Nutritional Risk Index; NYHA—New York Heart Association; eGFR—estimated glomerular filtration rate; CRP—C-reactive protein; TP—total protein.

**Table 3:** Main parameters according to LVEF classification.

	HFrEF + HFmrEF (*n* = 24)	HFpEF (*n* = 62)	*p*-Value
Admission BI (score)	39.6 ± 25.3	44.6 ± 26.1	*p* = 0.4230
Discharge BI (score)	65.0 (45.0, 78.8)	70.0 (50.0, 90.0)	*p* = 0.2800
GNRI	86.1 ± 6.8	88.2 ± 9.9	*p* = 0.2820

LVEF—left ventricular ejection fraction; HFrEF—heart failure with reduced ejection fraction; HFmrEF—heart failure with mid-range ejection fraction; HFpEF—heart failure with preserved ejection fraction; BI—Barthel Index; GNRI—Geriatric Nutritional Risk Index.

## Data Availability

The data presented in this study are available upon request from the corresponding author.
